# Training scholars in dissemination and implementation research for cancer prevention and control: a mentored approach

**DOI:** 10.1186/s13012-018-0711-3

**Published:** 2018-01-22

**Authors:** Margaret Padek, Nageen Mir, Rebekah R. Jacob, David A. Chambers, Maureen Dobbins, Karen M. Emmons, Jon Kerner, Shiriki Kumanyika, Christine Pfund, Enola K. Proctor, Kurt C. Stange, Ross C. Brownson

**Affiliations:** 1 Prevention Research Center in St. Louis, The Brown School at Washington University in St. Louis, 1 Brookings Drive. Campus Box 1196, St. Louis, MO 63130 USA; 2 Division of Public Health Sciences, Department of Surgery, Washington University in St. Louis School of Medicine, 660 S. Euclid Ave. Campus Box 8100, St. Louis, MO 63110 USA; 3 Division of Cancer Control and Population Sciences, National Cancer Institute, Rockville, MD 20850 USA; 4 School of Nursing, National Collaborating Centre for Methods and Tools, McMaster University, 175 Longwood Road South, Suite 210a, Hamilton, ON L8P 0A1 Canada; 5 Harvard T.H. Chan School of Public Health, 677 Huntington Avenue, Kresge 1005, Boston, MA 02115 USA; 6 Canadian Partnership Against Cancer, 6202 Newburn Drive, Bethesda, MD 20816 USA; 7 Drexel University Dornsife School of Public Health, Philadelphia, PA 19104 USA; 8 Center for the Improvement of Mentored Experiences in Research, Wisconsin Center for Education Research, Institute for Clinical and Translational Research, School of Medicine and Public Health, University of Wisconsin-Madison, Madison, WI 53706 USA; 9 Center for Mental Health Services Research, The Brown School at Washington University in St. Louis, St. Louis, MO 63130 USA; 10 Center for Community Health Integration and the Case Comprehensive Cancer Center, 11000 Cedar Ave., Suite 402, Cleveland, OH 44106-7136 USA; 11 Division of Public Health Sciences and Alvin J. Siteman Cancer Center, Department of Surgery, Washington University School of Medicine, Washington University in St. Louis, St. Louis, USA

**Keywords:** Dissemination, Implementation, Training, Mentoring

## Abstract

**Background:**

As the field of D&I (dissemination and implementation) science grows to meet the need for more effective and timely applications of research findings in routine practice, the demand for formalized training programs has increased concurrently. The Mentored Training for Dissemination and Implementation Research in Cancer (MT-DIRC) Program aims to build capacity in the cancer control D&I research workforce, especially among early career researchers. This paper outlines the various components of the program and reports results of systematic evaluations to ascertain its effectiveness.

**Methods:**

Essential features of the program include selection of early career fellows or more experienced investigators with a focus relevant to cancer control transitioning to a D&I research focus, a 5-day intensive training institute, ongoing peer and senior mentoring, mentored planning and work on a D&I research proposal or project, limited pilot funding, and training and ongoing improvement activities for mentors. The core faculty and staff members of the MT-DIRC program gathered baseline and ongoing evaluation data regarding D&I skill acquisition and mentoring competency through participant surveys and analyzed it by iterative collective reflection.

**Results:**

A majority (79%) of fellows are female, assistant professors (55%); 59% are in allied health disciplines, and 48% focus on cancer prevention research. Forty-three D&I research competencies were assessed; all improved from baseline to 6 and 18 months. These effects were apparent across beginner, intermediate, and advanced initial D&I competency levels and across the competency domains. Mentoring competency was rated very highly by the fellows––higher than rated by the mentors themselves. The importance of different mentoring activities, as rated by the fellows, was generally congruent with their satisfaction with the activities, with the exception of relatively greater satisfaction with the degree of emotional support and relatively lower satisfaction for skill building and opportunity initially.

**Conclusions:**

These first years of MT-DIRC demonstrated the program’s ability to attract, engage, and improve fellows’ competencies and skills and implement a multicomponent mentoring program that was well received. This account of the program can serve as a basis for potential replication and evolution of this model in training future D&I science researchers.

**Electronic supplementary material:**

The online version of this article (10.1186/s13012-018-0711-3) contains supplementary material, which is available to authorized users.

## Background

Awareness of the need for scientific approaches to dissemination and implementation (D&I), to provide for more effective and timely ways to translate research in practical applications, has increased over the past two decades [1, 2]. With much of the cancer burden being preventable [3], the need to close the estimated 17-year gap between publication of new research findings and their application to prevention in practice has become urgent [4–6]. This increased awareness, and the development of D&I science has initiated programs training researchers in this domain [1, 7–9]. Funding agencies are designating funds for D&I specific proposals [10, 11], and there are increasing numbers of faculty positions focusing on D&I research [12]; however, the availability of training programs has lagged behind the demand from a growing D&I research workforce [13, 14]. Moreover, beyond efforts to identify requisite competencies, curricula, and desired outcomes of trainings, competencies must be put into action and tested [4–6, 15, 16].

The Mentored Training for Dissemination and Implementation Research in Cancer (MT-DIRC) program is perhaps the first to incorporate systematic mentored training for D&I research with a focus on cancer control. It builds on experiences with D&I science training programs of varying depth and formats offered by several organizations in the USA and globally [1, 17], with a particular focus on providing extended mentoring. Programs specific to mentoring in D&I science include the Implementation Research Institute (IRI) [8] sponsored by the National Institute of Mental Health (NIMH), and the Training Institute for Dissemination and Implementation Research in Health (TIDIRH), sponsored by the National Institutes of Health [7, 18]. IRI, which pioneered the approach of providing one-on-one 2-year mentoring, focuses on mental health researchers. The IRI recently added a component of training fellows to become mentors. TIDIRH, initially started as a 5-day training institute [7], has evolved into an online-based program with a 2-day in-person session. Both IRI and TIDIRH have completed 7 years of summer trainings. The TIDIRH program has an informal mentoring component, but there is no established expectation for those mentoring relationships to last beyond the few in-person sessions. Outside the USA, Knowledge Translation Canada (or more informally known as KT Canada) has also offered summer training institutes that rotate cities and hosts throughout the years [14, 19], and the Cochrane Collaboration, in conjunction with Public Health Insight at the University of Melbourne, offers a 1-day Knowledge Translation Training workshops [20]. Overall, relatively few of these programs provide significant opportunities for interaction or offer a sustained mentoring component [17].

Several universities offer ongoing support and training through their respective Clinical and Translational Science Award Programs [14, 21] as well as concentrations in implementation science as a component of their masters, doctoral, or post-doctoral programs [14, 16]. Online webinars and resources are also provided through institutions such as National Cancer Institute’s Division of Cancer Control and Population Science [22], the Veterans Affairs’ Quality Enhancement Research Initiative & Health Services Research & Development group through the Center for Implementation Practice and Research Support [23]. The variety in formats can be helpful to provide access for researchers who otherwise would not have the time or resources to be trained.

Various groups have undertaken work to develop competency and curriculum lists and expectations [12–16]. A few programs have conducted evaluations of their trainings pertaining to knowledge acquisition [13, 24], although few have assessed skill gains of their trainees over a longer period of time. An evaluation of the IRI using social network analysis found that mentoring was significantly related to outcomes, 2 years later, of collaborations focused on new research, grant submission, and scholarly publications [25]. The *Implementation Science* Editors’ recently issued call to action to build capacity for researchers to be able to conduct D&I research [17]. In this context, we set out to explore the effectiveness of the MT-DIRC D&I training program.

### Purpose of the MT-DIRC training program

Funded by the National Cancer Institute, MT-DIRC is an R25 grant-funded, post-doctoral education program, aimed at building capacity among early to mid-career, cancer control researchers in D&I science through supplemental training and mentorship in D&I research. During each year of the 2-year MT-DIRC program, fellows attended a 5-day summer institute at Washington University in St. Louis to receive didactic, group, and individual instruction on their research area of interest as it pertains to D&I science. Ongoing mentoring relationships occurred over the 2 years of the program, complemented by intermittent webinar sessions with topics chosen by the fellows themselves. This paper describes the various components of the MT-DIRC program and shares preliminary results from the first two trainee cohorts. The purpose of this paper is to describe the development of the MT-DIRC program and share the experience to date to inform the development of other D&I training programs with the aim of advancing the field of D&I research within cancer control and more broadly.

## Methods

### MT-DIRC faculty

The program was led by Program Director, Ross Brownson, a D&I scholar who has been a contributor to other similar D&I training programs (e.g., IRI and TIDIRH). Dr. Brownson was joined by other renowned D&I scholars (Table 1) who together have a combined 340-year experience with grant application writing, reviewing, and conducting D&I research [5] and have been central in setting the research agenda and priority for D&I science in the USA and Canada. This core faculty’s expertise covered all areas of the cancer control continuum as well as the various areas of D&I science. The core faculty members contributed to all areas of the program such as mentoring fellows throughout the year, shaping the agenda for the summer institute, and presenting at least one session. Core faculty are designated as co-investigators or consultants on this project and are compensated for their time accordingly. Three to four guest faculty members are invited to lecture on topics which enhance the breadth of expertise already available through the team of faculty. The guest faculty attended the summer institute anywhere from 1 day to the entire week depending on their schedules and provided fellows with special consultation in their particular subject area. The guest faculty members were given an honorarium for their time at the institute.

**Table 1:** Core faculty and staff

Name	Role	Institution	Discipline	Grant years served as core faculty
Ross Brownson	Mentor/PI	Washington University	Epidemiology	Years 1–5
Enola Proctor	Mentor	Washington University	Social work	Years 1–5
Graham Colditz	Mentor	Washington University	Epidemiology, medicine	Years 1–5
Matthew Kreuter	Mentor	Washington University	Health communications	Year 1–3
Maureen Dobbins	Mentor	McMaster University	Nursing	Year 1–5
Jon Kerner	Mentor	Canadian Partnership Against Cancer	Community psychology, epidemiology	Year 2–5
Anne Sales	Mentor	Ann Arbor VA	Nursing, health services research	Years 1–5
Christine Pfund	Mentor Consultant	University of Wisconsin Madison	Research mentoring	Years 1–5
Karen Emmons	Mentor	Harvard University	Public health, health behavior change	Years 2–4
Kurt Stange	Mentor	Case Western University	Medicine, public health	Years 2–4
David Chambers	Mentor	National Cancer Institute	Organizational behavior	Years 3–5
Shiriki Kumanyika	Mentor	Drexel University	Public health nutrition, epidemiology	Years 3–5
Debra Haire-Joshu	Mentor	Washington University	Public health, health behavior	Years 4–5
Maggie Padek	Program Coordinator	Washington University	Public health, social work	Years 1–5
Rebekah Jacob	Program Coordinator	Washington University	Public health, social work	Year 4–5

### Fellows

#### Eligibility

The MT-DIRC program sought a range of fellows from across the USA and internationally to apply for the program. In order to be considered for the fellowship, applicants must have completed a doctoral-level degree and have a full-time appointment in a research setting prior to applying. All researchers whose work focused along the cancer control continuum were welcome to apply [26]. The program focused on training early-career researchers; however, mid-to-late career researchers who were looking to shift their work to focus on D&I science were also encouraged to apply. Applicants were required to fill out an informational cover page, submit a two-page concept paper outlining a D&I pilot study, include an NIH-formatted biosketch, and two letters of references.

#### Recruitment

To recruit fellows, emails were sent out through various listservs and networks related to D&I science and cancer control. Flyers were made available at the annual Conference on the Science of Dissemination and Implementation each December in Washington D.C. Faculty and fellows were also encouraged to mention the program when giving presentations on their D&I work at related conferences.

#### Selection

In each year, every core faculty member reviewed a subset of the submitted applications, with each application reviewed by three faculty members. Faculty scored each application based on eight questions regarding overall quality, demonstrated commitment to D&I science, demonstration of experience working in trans-disciplinary networks, evidence of research support and potential, likelihood for career development, appropriate methods in concept paper, appropriate topic in concept paper, and potential impact of the work proposed. The scores were compiled and averaged with additional commentary included. All core faculty met by conference call to review scores and select fellows for that year’s cohort.

#### Award

Each year of the grant, MT-DIRC sponsored 12 fellows covering all costs for travel to St. Louis, MO, during the 5-day summer institute, hotel accommodations, and meals for the week as well as an additional $1000 for pilot funds to be used during the first year of the program. An additional collaboration with the Veterans Administration provided funding for two additional fellows who are VA-affiliated researchers. The Cancer Research Network also sponsored one additional fellow in the 2014 and 2015 cohorts.

### Components

**Fig. 1:**
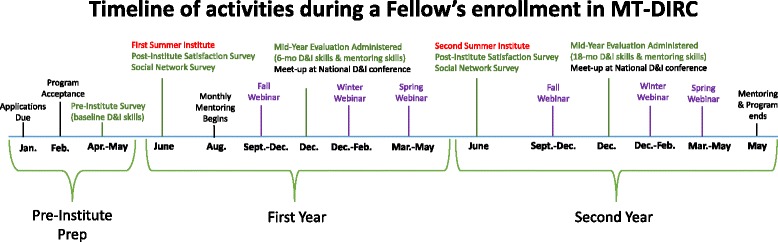
Timeline and components of MT-DIRC program

Figure 1 provides a visual depiction of the various components of the MT-DIRC program and their timing during the 2-year enrollment, including the points at which the baseline, 6- and 18-month D&I and Mentoring Competency Assessments were administered. A description of each component follows.

#### Competencies for MT-DIRC program curriculum

The foundation for the MT-DIRC program curriculum lies within a set of comprehensive D&I competencies. Building on prior work to define competencies for D&I science training [16, 19, 27], MT-DIRC faculty and staff engaged in a further effort to develop a comprehensive set of competencies that could be adapted to various types of curricula. This was done before the first summer institute in 2014. As described in detail elsewhere [15], a card-sorting activity was utilized and a list of 43 unique competencies was developed, sorted into learning levels (beginner, intermediate, and advanced), and used to guide the content that would be covered during the week-long summer institute.

To identify any remaining gaps within this curriculum, the MT-DIRC team conducted a concept mapping activity [28]. Concept mapping looked at competency development not only from the perspective of the researchers but also the practitioners, who are end users of the research. Nine clusters of competency themes were identified and mapped to the previous list of competencies to see where our previous competencies to assess alignment. See Tabak et al. [6] for further details.

#### Summer institute

Starting in 2014, 5-day MT-DIRC summer institutes have been held each year at Washington University in St. Louis. The institutes consisted of didactic sessions, one-on-one mentoring, and small group breakout sessions. The focus of the week was to address each of the identified competencies with the goals of improving D&I research skills and provide hands-on support to each fellow’s project development. Each day had a specific focus (e.g., aims, study design, and measurement) for project development. First-year fellows were asked to bring an updated version of the two-page D&I concept paper they had submitted in their application to the program. Returning fellows were asked to bring one or more “works in progress” to focus on during the week, such as in-progress manuscripts, grants in-development, and data sets. During breakout sessions, fellows took turns presenting their work and receiving feedback from mentors and peers. Breakout sessions were also used to allow work with their assigned MT-DIRC mentors, see Additional file 1 for a sample agenda of the summer institute.

#### Mentoring

All MT-DIRC fellows were matched with a core faculty mentor who was committed to working with 2–4 fellows across a 2-year time frame. Fellows were matched to their assigned mentor about 1 month prior to their arrival at the summer institute. Mentoring matches were done by the Program Coordinator with input from the faculty on preference, based on shared research interests or experiences.

In preparation, all faculty who served as mentors engaged in mentor training which was offered in three-stages by Dr. Christine Pfund (CP), Mentor Consultant, from the University of Wisconsin-Madison. The training included an asynchronous, self-paced component [29], a synchronous online component [30–33], and a face-to-face training at the summer institute. All mentors were required to participate in all three parts of the training. Table 2 provides an outline of the components of the mentor training as well as description of each.

**Table 2:** Mentor training components

Training activity	Description	Length/ format	Timing
Asynchronous online module	University of Minnesota’s CTSI online mentor training modules: http://www.ctsi.umn.edu/education-and-training/mentoring/mentor-training Modules explore mentoring models, mentor roles and responsibilities, structure and dynamics of the mentoring relationship, and strategies for facilitating and addressing challenges to the mentoring process [29]	90–120 min, self-paced	2–3 months before initial fellow assignment
Synchronous online meeting	Led by Mentoring Consultant. This included the introduction of a template mentoring contract for mentors to use with their fellows. This training was based on the evidence-based curriculum, Entering Mentoring [30–33]	60 min, online platform (e.g., Blackboard classroom, adobe connect)	1 month before training institute
Face-face meeting	Led by Mentoring Consultant: Review the previous mentor training sessions and review the institute agenda with a focus on the mentoring activities and discussed effective communication strategies	60 min, in person	Morning, day 1 of Summer Institute
Follow-up	During monthly core faculty calls, Mentoring Consultant would help troubleshoot any on-going mentoring concerns.	Varied	As needed

The Mentor Consultant provided continual support on the mentoring relationships throughout the duration of the program. Mentors were expected to have at least an hour of monthly contact with each of their assigned fellows (group mentor sessions could count toward that 1-hour requirement). If this expectation was not met, fellows or mentors were encouraged to contact the Program Coordinator and Principal Investigator so the situation could be addressed appropriately. On monthly calls with the core faculty, the group was invited to share facilitators or barriers to their current mentoring relationships. The core faculty tried to troubleshoot current issues as they arose as well as to provide suggestions on how to better facilitate these relationships.

#### Training of fellows

On the first day of the MT-DIRC institute, the Mentor Consultant presented a 1-hour session on “Evidence-Informed Mentoring for D&I Research” which included presentations of the key elements of research mentor training, background on the mentoring competencies, and overall expectations for the mentoring relationships, which are a critical element of the program. During this session, fellows were given time to identify their expectations for their relationships with MT-DIRC program mentors. Mentors and fellows were then given an opportunity to meet for the first time and lay out expectations for the week and beyond. Mentors and fellows had face-to face contact every day during the institute, and on the final day were given the opportunity to plan how the mentoring relationship would be handled during the monthly calls and the 2 years of the fellowship.

#### Ongoing interactions with fellows through calls and webinars

Since most of the mentoring interaction between the fellows and faculty mentors was conducted at a distance, the team brainstormed ways for the entire cohort to connect between their attendance at the two summer institutes. While there were informal gatherings at the Annual Conference on the Science of Dissemination and Implementation in Health [34] held in each December in Washington D.C., fellows indicated early on in the program a preference for continuing education throughout the year. The team at first addressed this with quarterly cohort calls for the fellows. Beginning in 2015, quarterly webinars were instituted. These webinars were generally led by one or two faculty members on a topic of interest to the group. Webinar attendance was limited to MT-DIRC fellows past and present. Webinars were 1 h in length and consisted of a formal presentation portion with time for questions and answers at the end. As of January 2018, the program had hosted seven webinars on a variety of topics that included how to craft an aims page, demystifying the grant review process, funding mechanisms, career awards, and mixed methods evaluation and building D&I research programs and choosing models and frameworks. All webinars are archived and available to all fellows and faculty. These webinars complemented other ongoing distance education training activities such as those offered by the NCI and the VA [35, 36].

#### Pilot funds

To assist fellows in securing grants, the program offered $1000 in pilot funds to each fellow during their first year in the program. These funds were intended to support pilot research with the goal of helping fellows obtain preliminary results which could be leveraged for larger D&I grant applications. Fellows also used these funds to attend and present at various conferences, attend relevant meetings pertaining to D&I science, and support the effort of research assistants or a data analyst, or for participant incentives in pilot work.

### Evaluation

To assess the on-going mentoring relationship, D&I skill level, as well as the general satisfaction with the institute and the MT-DIRC program overall, a variety of surveys were conducted throughout the year via the Qualtrics online survey platform. In addition to the various surveys, fellows’ academic outputs (grant applications submitted and awarded, publications, and presentations) were tracked over the years. The project coordinator utilized these outputs for annual NCI training grant reporting purposes and the long-term assessment of fellows’ productivity.

#### Pre-institute skills survey

A pre-institute survey was administered to fellows 1 month before they attended their first summer institute. This 20-min survey was used to collect pertinent demographic information, intended for grant reporting purposes as well as some logistical questions in preparation of the training institute. The survey was also used to collect baseline data on each fellow’s level of D&I skills according to the 43 competencies (a full list of competencies is included in Additional file 1). Fellows were also asked to indicate their general mentoring needs. This survey was administered only once at the beginning of each fellow’s time in the program.

#### Post-institute satisfaction survey

At the completion of the 5-day institute, fellows were immediately sent an online survey asking them to rate the various components of the institute. Fellows rated their satisfaction with information communicated to them before the institute, travel and accommodation arrangements, quality of individual faculty presentations, structure of the institute’s agenda, and any additional feedback regarding the institute.

#### Six and 18-month post-institute skills and mentoring survey

Longitudinal data were collected via survey from fellows at 6 and 18 months after attending their first institute. Fellows were asked to reassess their D&I skill levels for the same 43 competencies that were assessed in the pre-institute survey. Consistent with other literature on skill assessments, we describe these outcomes as skills, although there are some overlaps with self-efficacy [37–39]. In addition, theoretical concepts around self-efficacy posit that researchers can have the same knowledge or skills rating but differ in their self-efficacy, thus differ in their implementation of that knowledge and those skills [37, 38]. The 6- and 18-month post-institute surveys also assessed fellows’ mentoring relationships. Fellows were asked to rate the skills of their mentors utilizing the Mentoring Competency Assessment (MCA). The MCA is a validated [39] 26-item skill inventory evaluating six areas of mentoring competencies: maintaining effective communication, aligning expectations, assessing understanding, addressing diversity, fostering independence, and promoting professional development [39].

To gain additional evaluation of the program’s performance, fellows were asked to rank their mentoring priorities and their satisfaction with those priorities being met. They were asked to rate (on a five-point Likert scale) the importance of eight mentoring priority areas (skill building, sharing resources and infrastructure, performance feedback, providing opportunity, career planning, professional networking, professional socialization, providing emotional support) and their level of satisfaction with each of the aforementioned priorities being met.

As of this writing, all four cohorts had completed their pre-institute survey; three cohorts had completed the 6-month post-institute survey, and two had completed the 18-month post-institute evaluation. We are reporting these preliminary data at this time to make them accessible to others attempting to replicate a similar program.

#### Data analysis

Using descriptive statistics, we examined the demographic composition of the four cohorts. D&I skills and mentoring outcomes were analyzed for the two cohorts (2014 and 2015 cohorts) with complete data (pre-institute, 6- and 18-month post-institute surveys completed). D&I skill gains were tested using a Repeated Measures ANOVA between the three time points (pre-institute, 6- and 18-month post-institute) with Greehouse-Geisser’s correction utilized where assumption of sphericity was violated [40]. Cohen’s D between the 18-month post-institute mean and the pre-institute mean was used to determine overall effect size. Cohen suggests the following effect ranges: small 0.2, medium 0.4, and large 0.8 [40]. In keeping with the work that was done with the card-sorting to develop these competencies [15], a summary variable was created to look at the competency scores by each domain and each competency skill level. For mentoring competencies, independent *t* tests were used to compare mentors’ self-ratings versus fellows’ ratings of mentors’ competencies across the MCA. The analysis looked at the fellows and mentor’s initial 6-month assessment of mentoring skills. Additionally, differences in mean ratings of importance and satisfaction among the eight mentoring priority areas were explored. These measurements were taken at fellows’ 6- and 18-month post-institute survey.

## Results

### Fellows

From 2014 to 2017, 56 fellows participated in the MT-DIRC program. Participants were recruited nationally with the most successful recruitment tactic being word of mouth from colleagues in a previous cohort. Demographics for each of the four participating cohorts are in Table 3. A majority (79%) of the fellows accepted into the program were female, focused their research on Cancer Prevention (48%) and were Assistant Professors (55%). A majority (59%) came from an allied health discipline. Fellows represented all geographic regions of the USA, two fellows were from Canada, and three were from Australia. An additional three fellows indicated “other” as they were foreign-nationals currently employed in the USA. All fellows attended the institute and on average, 15–20 fellows from all of the cohorts attended each webinar.

**Table 3:** Fellow demographics

Fellow cohorts (N)					
	2014 (13)	2015 (15)	2016 (14)	2017 (14)	Total (56) %
Demographics					
Male	2	4	4	2	12 (21%)
Female	11	11	10	12	45 (79%)
Area of cancer control					
Prevention	8	3	7	9	27 (48%)
Detection	1	1	1	1	4 (7%)
Diagnosis	0	2	1	0	3 (5%)
Treatment	0	3	4	4	11 (20%)
Survivorship	4	6	1	0	11 (20%)
Discipline					
Allied health	10	7	8	8	33 (59%)
Social science	1	0	2	2	5 (9%)
Basic science	1	0	0	0	1 (1%)
Clinical	1	8	4	4	17 (31%)
Position					
Postdoctoral researcher	4	1	1	2	8 (14%)
Research scientist	2	2	0	0	4 (7%)
Assistant professor	4	8	11	8	31 (55%)
Associate professor	3	3	1	2	9 (16%)
Professor	0	1	1	1	3 (6%)
Other	0	0	0	1	1 (2%)
Nationality					
USA	12	12	13	11	48 (86%)
Canada	0	1	0	1	2 (4%)
Australia	0	2	1	0	3 (5%)
Other	1	0	0	2	3 (5%)

### D&I skills

A primary goal of the MT-DIRC program is to increase the skills of D&I research across a range of competencies identified by this program. Results indicate that fellows have had a statistically significant improvement across all 43 targeted competencies and that these gains are sustained at the 18-month time point. Mean skills gain score at the baseline, 6- and 18-month time points, grouped by competency domains are presented in Table 4. The skill that saw the largest change between pre- and 18 months was “Identify common D&I measures and analytic strategies for your research questions” (MD = − 1.69, Cohen’s *D* = 2.151). The skill that saw the least change between pre- and 18 months was “Identify sites to participate in D&I studied and negotiate or proved incentives to secure their involvement” (MD = − 0.92, *D* = 0.88). There were improvements in all summary domains. The domain that saw the largest increase in skills between pre- and 18 months was “Definitions, Background and Rationale” (MD = − 1.22, *D* = 1.97). The domain that saw the least increase in skill between pre- and 18 months was “Design and analysis” (MD = − 1.27, *D* = 1.37).

**Table 4:** Changes in fellows’ (*n* = 26) D&I skills over time grouped by summary competency domain

	Mean and standard deviation			Mean difference and Cohen’s *D*			Repeated-measures ANOVA
Competency domains	Pre	6 month	18 month	Pre-6 month	6–18 month	Pre-18 month	*F* value
A: Definitions, background, and rationale	2.80 ± 0.67	3.69 ± 0.56	4.03 ± 0.51	− 0.89*** *d* = 1.43	− 0.34** *d* = 0.63	− 1.22*** *d* = 1.97	54.27***
B: Theory and approach	2.57 ± 0.76	3.42 ± 0.70	3.78 ± 0.61	− 0.86*** *d* = 1.16	− 0.35*** *d* = 0.55	− 1.21*** *d* = 1.76	66.97***
C: Design and analysis	2.38 ± 0.69	3.28 ± 0.63	3.65 ± 0.64	− 0.90*** *d* = 1.37	− 0.37** *d* = 0.59	− 1.27*** *d* = 1.37	57.0***
D: Practice Based Considerations	2.75 ± 0.76	3.61 ± 0.61	3.91 ± 0.70	− 0.86*** *d* = 1.25	− 0.31* *d* = 0.47	− 1.17*** *d* = 1.60	44.06***

Scale: (*1* not at all skilled, *5* extremely skilled)

*Note:* Greenhouse Geiser-corrected *F* statistic shown where sphericity was violated

*Indicates significance reached at *p* ≤ .05

**Indicates significance reached at *p* ≤ .01

***Indicates significance reached at *p* ≤ .001

Changes in skill level across the competency domains were also analyzed using the skill levels that were predetermined in the card sort activity [15]. Among the three competency categories (beginner, intermediate, advanced), the largest increase in skill was shown for beginner skills (at 18 months, MD = − 1.26, *D* = 2.10) (Table 5)**.**

**Table 5:** Changes in fellows’ D&I skills over time grouped by skill competency level (*n* = 26)

	Mean and standard deviation			Mean difference and Cohen’s *D*			Repeated-measures ANOVA
Individual competency level	Pre	6 month	18 month	Pre-6 month	6–18 month	Pre-18 month	*F* value
Beginner	2.98 ± 0.63	3.84 ± 0.63	4.23 ± 0.57	− 0.87*** *d* = 1.38	− 0.39*** *d* = 0.65	− 1.26*** *d* = 2.10	*F* = 73.56***
Intermediate	2.55 ± 0.67	3.41 ± 0.58	3.76 ± 0.56	− 0.87*** *d* = 1.38	− 0.35** *d* = 0.61	−1.21*** *d* = 1.95	*F* = 63.45***
Advanced	2.04 ± 0.66	3.04 ± 0.60	3.25 ± 0.85	− 0.99*** *d* = 1.58	− 0.21 *d* = 0.29	− 1.21*** *d* = 1.59	*F* = 34.17***

Scale: (*1* not at all skilled, *5* extremely skilled)

*Note:* Greenhouse Geiser-corrected *F* statistic shown where sphericity was violated

*Indicates significance reached at *p* ≤ .05

**Indicates significance reached at *p* ≤ .01

***Indicates significance reached at *p* ≤ .001

### Mentoring

Mentoring is a critical element of the MT-DIRC program, and therefore, the mentoring skills of the core faculty as well as the mentoring relationships were evaluated on several levels. Table 6 shows comparisons at 6 months between the ratings the 2014, 2015, and 2016 fellows gave their mentors and those the mentors gave themselves during the same period across the 26 items of the MCA [39]. Fellows and mentors have similar rating in six of the identified 26 competencies having shown no significant difference between the mean scores of mentors and fellows. The remaining 20 competences showed a significant difference in the way fellows rated their mentors and the way that mentors rated themselves.

**Table 6:** Mentoring Competency Assessment average ratings by mentors and cohorts 6-month post-institute

Competency	Fellows* (6 months) *M* (SD), *N* = 40	Mentors (6 months) *M* (SD), *N* = 11	Significance level (*p* = .050)
Maintaining effective communication			
Active listening	6.64 (0.53)	5.64 (0.92)	*p* = .000
Providing constructive feedback	6.46 (0.79)	5.73 (1.00)	*p* = .014
Developing a trusting relationship	6.40 (1.00)	5.73 (0.78)	*p* = .046
Accommodating communication styles	6.34 (0.80)	5.27 (0.90)	*p* = .001
Pursuing strategies to improve communication	6.00 (1.17)	5.09 (0.53)	*p* = .017
Coordinating with other mentors	5.95 (1.31)	3.88 (1.35)	*p* = .001
Aligning expectations			
Considering mentor-mentee differences	6.37 (0.96)	5.36 (1.02)	*p* = .006
Setting research goals	6.05 (0.98)	5.64 (1.20)	*p* = .246
Setting clear relationship expectations	6.03 (1.16)	5.18 (1.16)	*p* = .039
Developing strategies to meet goals	5.97 (1.16)	5.18 (1.07)	*p* = .051
Aligning expectations	5.94 (1.19)	5.27 (1.00)	*p* = .098
Assessing understanding			
Enhancing mentee skills	5.94 (0.99)	5.09 (0.83)	*p* = .014
Assessing mentee knowledge	5.82 (1.08)	5.36 (0.80)	*p* = .204
Estimating mentee ability	5.79 (1.03)	5.09 (1.04)	*p* = .058
Fostering independence			
Acknowledging mentee’s professional contributions	6.23 (1.00)	5.89 (1.26)	*p* = .387
Negotiating path to independence	6.07 (1.25)	4.80 (1.03)	*p* = .007
Building confidence	6.06 (1.17)	5.09 (1.13)	*p* = .020
Motivating mentees	5.97 (1.21)	5.09 (1.22)	*p* = .040
Stimulating creativity	5.86 (1.19)	4.73 (0.78)	*p* = .005
Addressing diversity			
Accounting for different backgrounds of mentors and mentees	6.68 (0.653)	5.20 (0.91)	*p* = .000
Accounting for biases and prejudices	6.00 (1.17)	5.00 (1.09)	*p* = .026
Promoting professional development			
Understanding impact as role model	6.10 (0.93)	4.73 (1.10)	*p* = .000
Helping mentees acquire resources	5.90 (1.04)	4.91 (1.04)	*p* = .010
Helping establish a work/life balance	5.84 (1.25)	4.70 (0.94)	*p* = .018
Setting career goals	5.81 (1.27)	4.91 (1.22)	*p* = .050
Helping network effectively	5.70 (1.26)	4.73 (1.48)	*p* = .044

*Note:* Means represent average rating on a Likert scale of 1 to 7 “least skilled…” to “most skilled…”?

*2014, 2015, and 2016 fellows

**Fig. 2:**
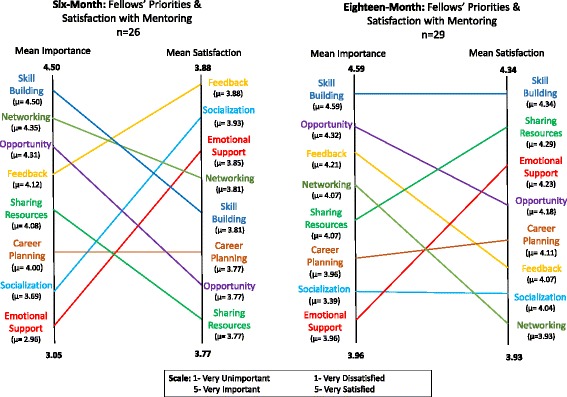
Fellow’s mentoring priorities and satisfaction at 6 and 18 months

Alignment of fellows’ priorities and their satisfaction of having those priorities met was assessed using five-point Likert scale about the mentoring priorities and satisfaction. This alignment was assessed at both the 6- and 18-month marks to explore how importance and satisfaction changed over time. As shown in Fig. 2, while there was general alignment between the fellows rating of importance for a given mentoring domain (priority) and their satisfaction with that needs being met; priorities and satisfaction did shift over their time in the program. Skill building was consistently seen as very important (mean importance, *μ* = 4.50), but fellows initially indicated a mismatch in rating their satisfaction with this need being met lower than other domains (mean satisfaction, *μ* = 3.81). However, by the 18-month point, there was alignment in ranking of fellows’ importance and satisfaction place on skill building. By the 18-month point, most of the factors had improved their ratings in terms of satisfaction to fellows and were more closely aligned to the initial fellows’ perceived importance in that mentoring area. Across the board, mentors and fellows indicated that dedicated time for each other was their biggest barrier to these relationships.

## Discussion

Overall, early results from the MT-DIRC program show fellows are improving in their D&I skills and mentoring needs of the fellows are being met. Findings, discussed below, highlight the impact of the program and provide guidance for similar model programs. The emphasis on mentoring and skill development throughout the course of the program we believe has been integral to its success. However, the team has aimed to keep the program flexible to meet the needs of each cohort and the individual fellows and do not believe that all programs must contain every component in order to be successful. Rather, those interested in creating similar programs can utilize these results to inform their decision-making and help shape the content and format for their programs.

### D&I skills

All D&I skills showed significant improvement from the time of entering into the program to the 6-month post-institute, with further improvements 18-month post-institute. While fellows showed improvement from their time in the program just within the first 6 months, there was continued improvement in skill levels over the subsequent 12 months suggesting that participants were using the skills they learned at the institute over time which led to continued growth in these areas.

Since the program was aimed at early-career researchers or mid-career researchers looking to switch their research focus into D&I science, it is not surprising that the largest area of improvement for skill levels were previously identified beginner skills (see Table 5 and Additional file 2). And since most of those beginner level skills were located within the “Definitions, Background, and Rationale” domain, we also saw that domain as having the largest overall skill increase. With the field of D&I research growing and its visibility expanding, more fellows are entering the program with a D&I conceptual framework to build upon when they enter the program and are able to master the beginner level concepts more quickly. These results may also suggest that the program does a reasonable job in training beginner level D&I skills, and more focus is needed on addressing advanced-level skills, whether through additional training throughout the year or perhaps even adding a training program in the future that focuses on advanced level D&I skills. However, the level of advancement of the D&I skills over the various time points also speaks well to the results of the original mapping of these competency levels when originally developed [15].

### Mentoring

Our findings show that, in general, faculty mentors rated themselves lower on mentoring skills (MCA) than their fellows rated them, consistent with previous research [39]. This finding may indicate that the standard mentors hold themselves to may be higher, in comparison to the expectations fellows have for a mentoring relationship in an academic research context. It may also show that the mentor training, while building mentor’s confidence in mentoring skills, also enabled mentors to more critically evaluate themselves [41]. Fellows, only having received a 1-h training related to mentoring, were perhaps less inclined to evaluate their mentors to the same degree due the narrower scope of training received regarding mentorship competencies. This comparison of mentor self-assessment and fellow assessment allows for identification of future areas to focus on within our program, either by providing feedback for changes to the program structure or greater support for mentors.

While this is a formal mentoring program, mentoring contracts with fellows were not required (though they were encouraged and a template was provided) nor were meeting duration or structures stipulated. The only requirement was that mentors connected with each of their fellows at least once per month, for a recommended time of 1 h. Mentors were given the choice to hold phone calls or skype calls (very few could arrange face to face meetings) and whether they wanted to conduct group calls with all their fellows and/or individual calls. These elements of flexibility may suggest that perceived capabilities of the mentor may vary based on the expectations set out from the initial contact between the mentor and fellow pair. As noted in other research, codifying the mentoring relationship roles, in a way in which calls upon fellows to also be active participants in articulating their needs, may enhance the quality of the relationship and improve perceived success of the relationship [42].

The questions in the Mentoring Priorities and Satisfaction section of the survey showed mismatches among the specific priority areas of skill building and sharing resources (see Fig. 2 for a visual depiction). This mismatch reflects that fellows were highly satisfied with these priority areas but considered them less important than other priority areas. Another mismatch at 6 months was the high importance of “skill building” (*μ* = 4.50) coupled with the lower satisfaction among fellows (*μ* = 3.81). This alignment levels out by the 18-month mark, suggesting that some skills and priorities may take more time to develop and conceptualize than others. However, during the 18-month assessment of satisfaction, the mean score of each priority area shifted upward, indicating a general positive shift in all areas of the mentor priority areas. So, even if there was no exact alignment of mentoring areas between priority and satisfaction, overall, the fellows indicated higher levels of satisfaction with their mentoring toward the end of their time in the program.

Other research has found that fellows desire greater emphasis within the mentoring relationship on broader topics such as personal-professional life balance, whereas our findings show that fellows typically rate these psychosocial topics as less important while being highly satisfied with the support in these topics [43]. In particular, “emotional support” is listed as the least important priority for fellows but is rated highly in terms of satisfaction. It may be that expectations in these areas are lower than that of others. Since the mentoring received here is of a formal nature and geared specifically toward D&I capacity-building in cancer research, it may be that the fellows are already receiving the psychosocial support they need from previously established mentoring relationships at their home institutions.

### Limitations

There are some limitations in the way that skill assessments were conducted. First, the assessments of fellows’ skills were self-rated. Fellows were asked about “How skilled do they feel” for a specific competency at that particular point in time. The project team has suggested administering an objective skill test to fellows at the end of their time in the program. However, there is currently no known validated, objective D&I research skill test.

The relationships between the fellows and their home mentors were also not explored in depth. All applicants to the program were asked to identify home mentors in their applications, but there was no formalized process to connect home mentors to the assigned MT-DIRC mentors. This would have been an interesting relationship to follow to see if it produced higher satisfaction with mentoring or increased D&I skills. There is also the issue of power dynamic when assessing mentor’s skills by the fellows. While individual mentoring data are never shared, fellows may be unlikely to rate their mentors too critically given that these mentors serve in this role outside their normal research duties.

### Future directions

While additional data will be gathered that may strengthen or modify these results, we see the need to present these early findings as soon as possible to help inform the next set of D&I research training programs in their development. Final data collection for all participating cohorts will be completed with the 18-month assessment in January 2019. At that time, our team will assess the overall impact the program has had on all 56 fellows who have been enrolled. Additional case studies and qualitative analysis will help provide more robust feedback on the overall impact the program has had on the individual fellows and their career trajectory. Following methods used in the Luke et al. [25] evaluation of the IRI, the team plans to continue to collect data regarding the fellow’s academic output as well as conduct a Social Network Analysis of fellows’ professional relationships. There are plans for future work to connect the outcomes between a fellow’s social connectedness and their skill level.

Additional work can also be done to compare the effectiveness between the different D&I program formats. While this program currently uses its own evaluation tools, these tools can be formalized to be useful across disciplines and formats. This additional comparison could provide support for the range in the types of D&I training formats and assess which ones are suited to particular populations (e.g., students, post-docs, assistant professors, tenured associate, or full professors) or learning styles.

The results from this program are valuable in demonstrating the effect and need for such training programs. The experience with MT-DIRC over the past few years provide a sound understanding of the landscape and a solid infrastructure that can be carried forward to fit with the new training models supported by sponsoring agencies. Results like these demonstrate promising evidence of the effectiveness of such mentored training programs and support the funding of such programs in the future. It is the hope that those who intend to replicate D&I trainings can apply many of the principles outlined to develop and evaluate related training programs.

## Conclusions

In the first several years of the Mentored Training in Dissemination and Implementation Research in Cancer program, we were able to demonstrate the effects of such a mentored approach to training program as evidenced by the increase in skill level over time and the satisfaction that fellows have demonstrated with their mentoring and the program overall. We hope that by disseminating our findings and lessons learned, other groups can replicate and improve upon this model in order to continue to train the next generation of D&I science researchers.

## Additional files

Additional file 1: Sample summer institute agenda. (PDF 587 kb)

Additional file 2: MT-DIRC D&I skills 2014 and 2015 full data. (PDF 440 kb)

## Abbreviations

D&I: Dissemination and Implementation
IRI: Implementation Research Institute
KT: Knowledge Translation
MCA: Mentoring Competency Assessment
MT-DIRC: Mentored Training for Dissemination and Implementation Research in Cancer
NIMH: National Institute for Mental Health
TIDIRH: Training Institute in Dissemination and Implementation Research in Health
