# Supplementary material for: Training scholars in dissemination and implementation research for cancer prevention and control: a mentored approach
Source: Implement Sci. 2018 Jan 22;13:18. doi: 10.1186/s13012-018-0711-3 (PMC5778694; doi:10.1186/s13012-018-0711-3)
Supplement: Supplementary file 1 — Sample summer institute agenda. (PDF 587 kb) [file 13012_2018_711_MOESM1_ESM.pdf]

Sample MT-DIRC Agenda

	Day 1
7:30am-8:30am	**Faculty Only: Mentor Breakfast**<br>Presenter: Mentoring Consultant
8:45am-10:00am	**Welcome/Introductions**<br>Presenter: PI & Program Coordinator
10:00am-10:15am	Break
10:15am-11:00am	**Evidence-Informed Mentoring: How to be a good mentee**<br>Presenter: Mentoring Consultant
11:00am-12:00pm	**BREAKOUTS**<br>**1st Year Fellows: Initial Meeting with Mentors**<br>Mentors & 1st year Fellows<br>**2nd Year Fellows: How to be an Evidence-Informed D&I Mentor**<br>Mentoring Consultant & 2nd year Fellows
12:00pm-1:00pm	**Lunch Consultations**
1:00pm-1:45pm	**How to Choose your Methods, Frameworks, Models**<br>Presenter: Guest Faculty
1:45pm-2:00pm	Break
2:00pm-3:30pm	**BREAKOUTS**<br>**1st Year Fellows: Present Concept Paper**<br>**2nd Year Fellows: Works in Progress**
3:30pm-4:30pm	**D&I resource of the Day**<br>Presenter: Core Faculty

	Day 2
9:00am-9:15am	**Plan for the Day**<br>Presenter: PI/Program Coordinator
9:15am-9:45am	**Building Partnerships and Sustainability for D&I Research**<br>Presenter: Core Faculty
9:45am-10:35am	**Stakeholder Engagement & Disparities Research Panel**<br>Presenter: Core & Guest Faculty
10:35am-10:50am	Break
10:50am-12:00pm	**BREAKOUTS**<br>**1st Year Fellows: Refining study aims**<br>**2nd Year Fellows: Works in Progress**
12:00pm-1:00pm	**Lunch Consultations**
1:00pm-1:50pm	**Disseminating Digital Health Innovations**<br>Presenter: Guest Faculty
1:50pm-2:00pm	Break
2:00pm-3:00pm	**BREAKOUTS**<br>**1st Year Fellows: Refining study aims**<br>**2nd Year Fellows: Works in Progress**
3:00pm-3:15pm	Break
3:15pm-4:15pm	**System Science and D&I research**<br>Presenter: Guest Faculty
4:15pm-4:45pm	**D&I Resource of the Day: Online Trainings & Toolkits**<br>Presenter: Core Faculty

	Day 3
9:00am-9:15am	**Plan for the Day**<br>Presenters: PI/ Program Coordinator
9:15am-10:00am	**Navigating the Politics of Innovation Implementation**<br>Presenter: Guest Faculty
10:00am-10:15am	Break
10:15am-11:00am	**Strategies & Measures for D&I research**<br>Presenters: Core Faculty
11:00am-12:15pm	**<u>BREAKOUTS</u>**<br>**1st Year Fellows: Refining your study design and analytic approaches**<br>**2nd Year Fellows: Peer Product Review**
12:15-1:15pm	**Lunch Consultations**
1:15pm-2:15pm	**D&I Grant Writing/Review Panel**<br>Presenters: Core Faculty
2:15pm-2:30pm	Break
2:30pm-3:45pm	**<u>BREAKOUTS</u>**<br>**1st Year Fellows: Refining your study design & analytic approaches**<br>**2nd Year Fellows: Peer Product Review**
3:45pm-4:30pm	**Mid-Week Mentor Groups Check-in**<br>All Core Faculty & Fellows

	Day 4
9:00am-9:15am	**Plan for the Day**<br>Presenters: PI/ Program Coordinator
9:15am-10:00am	**Budget Development and the Business side of D&I research**<br>Presenter: Core Faculty
10:00am-10:15am	Break
10:15am-12:00pm	**BREAKOUTS**<br>**1st Year Fellows: Fish Bowl: Presenting revised research plan**<br>**2nd Year Fellows: Provide peer feedback for 1st Year Fellows**
12:00pm-1:00pm	**Lunch Consultations**
1:00pm-1:45pm	**Shared Decision Making in D&I research**<br>Presenter: Guest Faculty
1:45pm-2:00pm	Break
2:00pm-2:45pm	**Reporting Contextual Factors/External Validity**<br>Presenter: Core Faculty
2:45pm-4:00pm	**BREAKOUTS**<br>**1st Year Fellows: Fish Bowl: Presenting revised research plan**<br>**2nd Year Fellows: Provide peer feedback for 1st Year Fellows**
4:00pm-5:00pm	**Getting your D&I paper published: Panel of Journal Editors and Authors**<br>Presenter: Core Faculty

	Day 5
8:45am-9:00am	**Plan for the Day**<br>Presenter: PI/Program Coordinator
9:00am-10:00am	**Responsible Conduct of Research: Concerns Specific to D&I research**<br>Presenter: Core Faculty
10:00am-10:15am	Break
10:15am- 11:00am	**Final Meeting with your Mentor**<br>1st & 2nd Years together
11:00am-12:00pm	**Final Wrap Up Discussion/Next Steps**<br>Presenter: PI and Program Coordinator

Note: Topics change each year depending on what was covered in year's past and the cohort of Fellows. Guest Faculty change each year and are invited based on topics to be covered. This agenda is meant to highlight the basic format of the training institute as well as a sample of topics that were covered in one institute.
