# Supplementary material for: Training scholars in dissemination and implementation research for cancer prevention and control: a mentored approach
Source: Implement Sci. 2018 Jan 22;13:18. doi: 10.1186/s13012-018-0711-3 (PMC5778694; doi:10.1186/s13012-018-0711-3)
Supplement: Supplementary file 2 — MT-DIRC D&I skills 2014 and 2015 full data. (PDF 440 kb) [file 13012_2018_711_MOESM2_ESM.pdf]

MT-DIRC D&I Skills 2014 & 2015 Full Data
N=26

Skill level	Competency	Pre Mean (Std)	6 Mo. Mean (Std)	18 Mo. Mean (Std)	Pre vs. 6 Mo. MD	6 Mo. vs 18 Mo. MD	Pre vs 18 Mo. MD	Cohen's *d* Pre to 18 Mo.	Maulchy 's* p<.05	Within-Subjects Effect**
**Domain A: Definitions, Background and Rationale**										
B	Define and communicate D&I research terminology	2.85 ±0.784	3.92 ±0.628	4.23 ±0.587	-1.077 p=.000	-0.308 p=.053	-1.077 p=.000	1.992	p=.038 έ= .808	df (1.616,40.389), F=45.2 p= 0.000
B	Define what is and what is not D&I research	2.96 ±0.824	3.92 ±0.720	4.23 ±0.587	-1.00 p=.000	-.269 p=.329	-1.269 p=.000	1.775	p=.302	df (2,50) , F= 28.028 p= 0.000
B	Differentiate between D&I research and other related areas such as efficacy research and effectiveness research	3.42 ±0.857	3.96 ±0.662	4.38 ±0.571	-.538 p=.007	-.423 p=.003	-.962 p=.000	1.318	p=.160	df (2,50), F=22.776 p= 0.000
B	Identify the potential impact of disseminating, implementing and sustaining effective interventions	3.12 ±0.909	4.00 ±0.632	4.31 ±0.679	-.885 p=.000	-.308 p=.053	-1.192 p=.000	1.483	p=.003 έ=.718	df(1.437, 35.915), F=24.011 p= 0.000
B	Describe the range of expertise needed to conduct D&I research	2.88 ±0.993	3.85 ±0.925	4.31 ±.736	-.962 p=.000	-.462 p=.034	-.1.142 p=.000	1.636	p=.144	df(2, 50), F = 13.126 p= .000
I	Determine which evidence-based interventions are worth disseminating and implementing	3.23 ±0.815	3.73 ±0.778	4.08 ±0.688	-.500 p=.020	-.346 p=.108	-.846 p=.000	1.137	p=.847	df(2, 50) = 13.126 p= 0.000
I	Assess, describe and qualify the context for effective D&I	2.62 ±0.941	3.46 ±0.761	4.08 ±0.744	-.846 p=.001	-.615 p=.020	-1.462 p=.000	1.721	p=.951	df(2,50), F = 24.419, p= 0.000
I	Identify existing gaps in D&I research	2.50 ±0.906	3.50 ±0.707	3.65 ±0.797	-1.00 p=.000	-.154 p=1.00	-1.154 P=.000	1.347	p=.683	df(2,50), F = 27.949, p= 0.000
I	Identify the potential impact of scaling down an ineffective but often used intervention	2.08 ±0.935	3.19 ±0.849	3.58 ±0.902	-1.115 p=.000	-.385 p= 0.171	-1.500 p=.000	1.632	p=.380	df(2,50), F = 25.927 p= 0.000
I	Formulate methods to address barriers of D&I research	2.38 ±0.898	3.31 ±0.788	3.42 ±0.758	-.923 p=.001	-.115 p=1.000	-1.038 p=.000	1.251	p=.197	df(2,50) F= 16.311, p= 0.000
**Domain B: Theory and Approaches**										
B	Describe a range of D&I strategies, models, and framework	2.62 ±0.804	3.69 ±0.838	4.08 ±0.796	-1.077 p=.000	-.385 p=.046	-1.462 p=.000	1.824	p=.861	df(2,50), F = 50.346, p= 0.000
I	Identify appropriate conceptual models, frameworks or program logic for D&I change	2.62 ±0.941	3.54 ±0.859	4.08 ±0.744	-.923 p=.000	-.538 p=.004	-1.462 p=.000	1.721	p=.107	df(2,50) F= 47.595 p= 0.000
I	Identify core elements of effective interventions and recognize risks of making modifications	2.69 ±0.736	3.35 ±0.797	3.54 ±0.582	-.654 p=.002	-.192 p=.608	-.846 p=.000	1.281	p=.655	df(2,50) F= 14.955 p= 0.000
I	Describe a process for designing for dissemination	2.77 ±0.908	3.46 ±0.859	3.81 ±0.749	-.692 p=.002	-.346 p=.029	-1.038 p=.000	1.249	p=.096	df(2,50), F= 21.641 p= 0.000
I	Describe the relationships between various organizational dimensions and D&I research	2.46 ±0.989	3.27 ±0.724	3.65 ±0.745	-.808 p=.000	-.385 p=.066	-1.192 p=.000	1.359	p=.554	df(2,50), F = 26.631 p= 0.000
I	Explain how knowledge from disciplines outside of	2.50 ±.949	3.38 ±0.804	3.62 ±.804	-.885 p=.000	-.231 p=.093	-1.115 p= .000	1.273	P=.018 έ=.779	df(1.557, 38.937), F=32.248

	health can help inform further trans disciplinary efforts in D&I									p= 0.000
I	Identify and articulate the interplay between policy and organizational process in D&I	2.31 ±1.08	3.31 ±0.884	3.69 ±0.838	-1.00 P=.000	-.385 P=.066	-1.385 P=.000	1.427	p=.191	df(2,50), F= 31.975, p= 0.000
**Domain C: Design & Analysis**										
B	Describe the core components of their external validity and their relevance to D&I research	2.81 ±0.939	3.50 ±0.860	4.08 ±0.796	-.692 p=.010	-.577 p= .002	-1.269 p= 0.000	1.459	p=.132	df(2,50), F= 23.507, p= 0.000
B	Identify common D&I measures & analytic strategies for your research questions	2.35 ±0.892	3.46 ±0.811	4.04 ±0.662	-1.115 p=.000	-.577 p= .000	-1.692 p= .000	2.151	p=.148	df(2,50), F = 62.025 p= 0.000
I	Identify and measure outcomes that matter to stakeholders, adopters, and implementers	2.92 ±0.891	3.62 ±0.804	4.00 ±0.748	-.692 p=.000	-.385 p=.046	-1.077 p=.000	1.312	p=.897	df(2,50), F = 26.678 p=0.000
I	Describe the application and integration of mixed-methods approaches in D&I research	3.23 ±0.908	3.85 ±0.925	4.15 ±0.834	-.615 p=.005	-.308 p=.218	-.923 p=.000	1.055	p=.787	df(2,50), F= 16.092 p= 0.000
I	Apply common D&I measures & analytic strategies for your research questions within your model/framework	2.50 ±0.812	3.46 ±0.761	3.85 ±0.732	-.962 p=.000	-.385 p=.028	-1.346 p=.000	1.746	p=.743	df(2,50), F= 43.605 p= 0.000
I	Identify possible methods to address external validity in study design reporting and implementation	2.27 ±0.778	3.15 ±0.732	3.69 ±0.618	-.885 p=.000	-.538 p=.004	-1.423 p=.000	2.021	p=.740	df(2,50), F = 39.126 p= 0.000
I	List the potential roles of mediators and moderators in a D&I study	2.46 ±0.905	3.31 ±0.788	3.73 ±0.724	-.846 p=.000	-4.23 p=.040	-1.269 p=.000	1.549	p=.737	df(2,50), F= 29.698 p= 0.000
I	Identify and articulate the trade-offs between a variety of different study designs for D&I research	2.19 ±0.895	3.35 ±0.846	3.69 ±0.788	-1.154 P=.000	-.346 P=.108	-1.500 P=.000	1.778	p=.353	df(2,50), F=37.726 p= 0.000
I	Describe how to frame and analyze the context of D&I as a complex system with interacting parts	2.31 ±0.970	3.04 ±0.774	3.62 ±0.852	-.731 p=.013	-.577 p=.014	-1.308 p=.000	1.434	p=.242	df(2,50), F= 21.203 p=0.000
I	Effectively integrate the concepts of sustainability/ sustainment and rational behind them in D&I study design	2.38 ±1.061	3.23 ±0.765	3.69 ±0.838	-.846 p=001	-4.62 p=.023	-1.308 p= .000	1.370	p=.142	df(2,50), F= 22.167 p=0.000
I	Describe gaps in D&I measurement and critically evaluate how to fill them	2.27 ±0.962	3.15 ±0.834	3.35 ±0.689	-.885 p=.000	-.192 p=.778	-1.077 p=.000	1.290	p=.078	df(2,50), F= 19.335 p= 0.000
A	Effectively explain and incorporate concepts of de-adoption and de-implementation into D&I study designs	1.80 ±0.866	3.08 ±0.812	3.20 ±0.913	-1.280 p=.000	-.120 p= 1.000	-1.400 p=.000	1.573	p=.221	df(2,50), F= 25.300 p= 0.000

MT-DIRC D&I Skills 2014 & 2015 Full Data
N=26

A	Incorporate methods of economic evaluation in D&I study design	1.81 ±0.749	2.77 ±0.815	2.85 ±1.120	-.962 p=.000	-.077 p= 1.000	-1.038 p= .000	1.091	p=.424	df(2,50), F= 21.847 p=0.000
A	Evaluate and refine innovative scale up and spread methods	2.00 ±0.938	2.92 ±0.628	3.19 ±1.021	-.923 p=.000	-.269 p= .387	-1.192 p=.000	1.213	p=.011 έ=.761	df(1.522, 38.042), F=19.805 p=0.000
**Domain D: Practice Based Considerations**										
B	Describe the importance of incorporating the perspective of different stakeholder groups	3.54 ±0.905	4.15 ±0.834	4.42 ±0.703	-.615 p=.005	-.269 p=.269	-.885 p=.000	1.085	p=.577	df(2,50), F= 16.063 p= 0.000
B	Describe the concept of measurement and fidelity	3.27 ±0.919	3.81 ±0.849	4.27 ±0.724	-.538 p=.040	-.462 p=.046	-1.000 p=.000	1.208	p=.737	df(2,50), F= 13.397 p=0.000
B	Articulate the strengths and weaknesses of participatory research in D&I research	2.92 ±0.891	3.96 ±0.916	4.19 ±0.895	-1.038 p=.000	-.231 p=.621	-1.269 p=.000	1.422	p=.951	df(2,50), F= 29.373 p= 0.000
I	Determine when engagement in participatory research in appropriate with D&I research	2.62 ±0.898	3.81 ±0.849	4.04 ±0.824	-1.192 p=.000	-.231 p= .555	-1.423 p= .000	1.647	p=.798	df(2,50), F= 41.364 p= 0.000
I	Describe the appropriate process for eliciting input from community-based practitioners for adapting an intervention	2.81 ±1.021	3.77 ±0.951	4.12 ±0.909	-.962 p=.000	-.346 p=.321	-1.308 p=.000	1.355	p=.593	df(2,50), F= 25.437 p= 0.000
I	Identify and apply techniques for stakeholder analysis and engagement when implementing evidence-based practices	2.38 ±0.941	3.38 ±0.804	3.77 ±0.908	-1.00 p=.000	-.385 p=.116	-1.385 p=.000	1.503	p=.762	df(2,50), F= 28.337 p= 0.000
I	Identify a process for adapting an intervention and how the process is relevant to D&I research	2.69 ±0.884	3.38 ±0.752	3.92 ±0.796	-.692 p=.002	-.538 p= .007	-1.231 p= .000	1.462	p=.505	df(2,50), F= 24.492 p=0.000
I	Explain how to maintain fidelity of original intervention during the adaption process	2.58 ±0.902	3.19 ±0.895	3.73 ±0.724	-.615 p=.005	-.538 p= .023	-1.154 p=.000	1.406	p=.912	df(2,50), F= 19.697 p= 0.000
I	Identify sites to participate in D&I studied and negotiate or proved incentives to secure their involvement	2.77 ±1.070	3.46 ±0.859	3.69 ±1.011	-.692 p=.005	-.231 p=.621	-.923 p=.000	0.883	p=.695	df(2,50), F=12.162 p= 0.000
I	Identify and develop sustainable partnerships for D&I research	2.73 ±1.002	3.65 ±0.892	3.73 ±0.874	-.923 p=.000	-.077 p= 1.000	-1.00 p=.000	1.063	p=.375	df(2,50), F= 20.933 p= 0.000
I	Describe how to measure successful partnerships for D&I research	2.12 ±0.952	3.31 ±0.788	3.31 ±0.970	-1.192 p=.000	.000 p=1.000	-1.192 p=.000	1.134	p=.256	df(2,50), F= 27.147 p=0.000
A	Use evidence and adapt D&I strategies for specific populations, settings, contexts, resources and/or capacities	2.54 ±0.989	3.38 ±0.804	3.77 ±0.908	-.846 p=.000	-.385 p=.090	-1.231 p=.000	1.295	p=.210	df(2,50), F= 22.686 p= 0.000

*Greenhouse Geiser Lower-bound= 0.5; Statistic is included if Mauchly's Test of Sphericity is significant

** correct F Stat is utilized based on Assumption of Sphericity

Skill Level: (B)= Beginner Skill, (I)= Intermediate Skill, (A)= Advanced Skill
